# In Vitro Sensitivity Test of *Fusarium* Species from Weeds and Non-Gramineous Plants to Triazole Fungicides

**DOI:** 10.3390/pathogens13020160

**Published:** 2024-02-10

**Authors:** Neringa Matelionienė, Renata Žvirdauskienė, Gražina Kadžienė, Evelina Zavtrikovienė, Skaidrė Supronienė

**Affiliations:** 1 Microbiology Laboratory, Institute of Agriculture, Lithuanian Research Centre for Agriculture and Forestry, Instituto al. 1, Akademija, LT-58344 Kedainiai, Lithuania; renata.zvirdauskiene@lammc.lt (R.Ž.); evelina.zavtrikoviene@lammc.lt (E.Z.); 2 Department of Soil and Crop Management, Institute of Agriculture, Lithuanian Research Centre for Agriculture and Forestry, Instituto al. 1, Akademija, LT-58344 Kedainiai, Lithuania; grazina.kadziene@lammc.lt

**Keywords:** fungicides, chemical control, *Fusarium*, mycelium growth inhibition, weed, non-gramineous plants, EC50

## Abstract

*Fusarium* species are common plant pathogens that cause serious crop losses worldwide. *Fusarium* spp. colonize not only the main host plants, crops, but also alternative hosts. The effectiveness of fungicide use in disease management ranges from very successful to possibly promoting the growth of the pathogen. Triazole fungicides are widely used to control these pathogens due to their broad-spectrum activity and systemic nature. This paper reviews the sensitivity of 40 *Fusarium* strains isolated from weeds, non-gramineous plants, and spring wheat to metconazole, prothioconazole, and tebuconazole. The effect of fungicides was determined by the percentage inhibition of *F. graminearum*, *F. culmorum*, *F. sporotrichioides*, and *F. avenaceum* fungal mycelial growth. The 50% effective concentration (EC50) values of all isolates on metconazole were lower than 2.9 mg L^−1^, prothioconazole EC50 ranged from 0.12 to 23.6 mg L^−1^, and tebuconazole ranged from 0.09 to 15.6 mg L^−1^. At 0.00025–0.025 mg L^−1^, the fungicides were ineffective, except for the growth of the *F. avenaceum* species. It was observed that isolates from weeds were more sensitive to low concentrations of fungicide than isolates from crop plants. In general, information is scarce regarding the comparison of fungicide resistance in *Fusarium* isolates from weed and crop plants, making this study an additional contribution to the existing knowledge base.

## 1. Introduction

Fusarium head blight (FHB), also known as scab, is a devastating disease of small-grain cereals worldwide [1]. This crop disease is still one of the main and economically most important diseases. *Fusarium* species affect a range of plant parts, such as roots, stems, and heads [2,3]. The most prevalent *Fusarium* species that cause FHB are *F. graminearum*, followed by *F. culmorum* and *F. avenaceum* [3]. These species are capable of producing mycotoxins such as zearalenone, deoxynivalenol (DON), T-2 toxin, and others, which reduce the quality and yield of the affected crops, making them unfit for human or animal consumption due to contamination with mycotoxins [4,5]. In Lithuania, *F. graminearum*, *F. culmorum*, *F. avenaceum*, and *F. sporotrichioides* are most associated with the disease [6]. Apart from the main host plants (wheat, barley, etc.), these pathogens are often found in other plants, such as crop weeds, grasses, and non-cultivated plants [7,8,9,10]. In order to ensure food safety and control economic loss, the management of FHB disease is a necessary aspect. Chemical control is increasingly being chosen for managing FHB disease due to the lack of disease-resistant varieties and effective biological control methods for the field [11].

In recent years, fungicides, such as triazoles, have been commonly used to control FHB by preventing the growth and spread of the *Fusarium* fungi [12,13]. Metconazole, prothioconazole, and tebuconazole are fungicides used in agriculture to control various fungal diseases, including FHB [14,15,16]. Triazole fungicides have been shown to be effective in inhibiting *Fusarium* species growth both in vitro and in vivo. In vitro studies have shown that triazole fungicides can inhibit the mycelial growth, spore germination, and toxin production in *Fusarium* species [17]. Through field trials, Paul et al. [18] showed that triazole-based fungicides are effective in controlling FHB and DON [19].

The effectiveness of triazole fungicides in inhibiting the growth of *Fusarium* species depends on several factors, including the type of fungicide, the timing and frequency of application, the susceptibility of the pathogen, and environmental conditions [15,20]. However, it has been extensively recorded and discussed that combining several management strategies was generally more effective against FHB disease severity than using just one strategy [12,21,22]. As well, the use of triazole fungicides can have potential risks, including the development of fungicide resistance, the accumulation of toxic residues in crops, and impacts on non-target organisms such as beneficial insects and soil microorganisms [23]. Since triazole fungicides have been used for controlling different pathogens on crops for long periods, some *Fusarium* species showed tolerance to these chemicals [24]. Despite this, it is not clear how *Fusarium* strains isolated from alternative plants (weeds), which, in our previous studies, showed the ability to cause very intense FHB disease [6] produce mycotoxins [25] and reduce grain yield [26], respond to fungicides. Finally, it is valuable to monitor the sensitivity of *Fusarium* populations from different sources to fungicides as part of integrated disease and weed management to implement control strategies.

This study aims to evaluate and compare the susceptibility of *Fusarium* species (*F. graminearum*, *F. culmorum*, *F. avenaceum*, and *F. sporotrichioides*) from weeds (scentless false mayweed, field pansy, shepherd’s purse, meadow grass, wild buckwheat) from crops (oilseed rape, field pea, sugar beet) and main host-plant spring wheat to triazole group fungicides (metconazole, prothioconazole, and tebuconazole) in vitro.

## 2. Materials and Methods

### 2.1. Isolation of Fusarium *Spp.* from Plants

The study was carried out at the Institute of Agriculture, Lithuanian Research Centre for Agriculture and Forestry, in 2021 and 2022. Plant samples were collected at the same time from five cropping system fields located in the middle of Lithuania (55°23′50″ N, 23°51′40″ E). Plants (crops and weeds) were collected during BBCH 65–79 (from the full flowering stage till the development of fruit), except sugar beet, which was taken at BBCH 38 (rosette growth stage). *Fusarium* strains were isolated from spring wheat (*Triticum aestivum*), field pea (*Pisum sativum*), sugar beet (*Beta vulgaris*), oilseed rape (*Brassica napus*), and weeds (*Viola arvensis*, *Fallopia convolvulus*, *Poa annua*, *Capsella bursa-pastoris*, and *Tripleurospermum inodorum*) according to [9]. *Fusarium* fungi were isolated from all morphological parts of the plant, including roots (r), crowns (c), stems (s), leaves (l), florets (fl), pods (p), and fruits (f). Only isolates from spring wheat were isolated from spikes. The plants were cut (1 cm in size) and sterilized for 3 min in 1% sodium hypochlorite (NaClO) solution and then rinsed 3 times in sterile distilled water (SDW) and dried on sterile filter paper in a laminar. Different parts of the plant were placed on potato dextrose agar (PDA, Merck) medium supplemented with 130 mg L^−1^ streptomycin sulfate, and the plates were incubated at 22 ± 2 °C in the dark for 2–4 days. The *Fusarium* fungi that appeared were purified via PDA and grown on a Spezieller Nährstoffarmer Agar medium (SNA) at 25 ± 2 °C for 10–30 days until spore mass formation. Single spores were picked and transferred onto PDA to obtain pure cultures for subsequent DNA extraction. Spore suspensions were prepared for further studies according to Suproniene et al. [9] and stored at −80 °C.

### 2.2. Identification of Fusarium *Spp.*

*Fusarium* species were isolated and identified by colony morphology and spore shape using a light microscope, as described by Leslie and Summerell [27]. DNA for PCR was extracted from 1- to 2-week-old mycelia using a ZR Fungal/Bacterial DNA MiniPrep kit (Zymo Research, Irvine, CA, USA) according to Sneideris et al. [28]. All morphologically identified cultures of *Fusarium* species were verified by species-specific PCR using the primer pairs (*F. avenaceum*: J1AF/R; *F. culmorum*: FC01F/R; *F. graminearum*: Fg16F/R; and *F. sporotrichioides*: AF330109CF/R) reported by Demeke et al. [29] and using conventional end-point PCR. The PCR reactions were performed in mixtures containing 2.5 μL of 10× PCR buffer (provided with the polymerase; Applied Biosystems, Waltham, MA, USA), 0.5 μL of dNTP Mix (10 mM each) (Thermo Fisher Scientific Baltics, Vilnius, Lithuania), 0.5 μL of each 25 μM primer, 1.25 U of AmpliTaq Gold polymerase (Applied Biosystems), 1 μL of extracted DNA template, and nuclease-free water up to a total volume of 25 μL. The thermocycling conditions consisted of initial denaturation and polymerase activation at 95 °C for 10 min; then, 38 cycles of 95 °C for 40 s, 55 to 62 °C for 30 s, and 72 °C for 55 s; followed by a final extension at 72 °C for 10 min. The annealing temperature was selected for each primer pair according to their original description [9,28,30]. 

*Fusarium* species were selected based on isolated species from the plant parts. However, we lost strains of *F. avenaceum* from oilseed rape, sugar beet, and field pea during storage and these were therefore not included. All *Fusarium* isolates and number per host plant are listed in Table 1. 

### 2.3. Preparation of Fungicide Solutions

Three different fungicides were used in this study: metconazole (Pestanal, Sigma Aldrich, JAV), prothioconazole (Carbosynth Limited, Dallas, TX, USA), and tebuconazole (Santa Cruz Biotechnology, Inc., San Diego, CA, USA). The initial fungicide solution was prepared with a concentration of 1000 mg L^−1^. Briefly, 10 mg of fungicide was weighed and dissolved in 0.5 mL of 70% ethanol. After dissolution, it was diluted with sterile distilled water to 10 mL. Tenfold dilutions in sterile distilled water were made from this initial fungicide stock solution.

The PDA (potato dextrose agar) medium was prepared according to the manufacturer’s instructions. The medium was prepared in 200 mL bottles, autoclaved at 121 °C 2 atm pressure for 20 min, and cooled in a water bath until 50 °C in temperature. After cooling, 1 mL of the prepared fungicide solutions of six concentrations and control-distilled water was added to the medium (with constant stirring). The final concentration gradients in the PDA media were: 0 (control), 0.00025; 0.0025; 0.025; 0.25; 2.5 and 25 mg L^−1^ of each fungicide. A medium with different concentrations of fungicides was poured under aseptic conditions into 90 mm diameter Petri dishes and left to solidify at room temperature. Each fungicide was tested against fungal species strains in four replicates. 

### 2.4. Mycelial Growth Inhibition Assay 

Sensitivity tests of thirteen *F. graminearum*, twelve *F. culmorum*, six *F. avenaceum*, and nine *F. sporotrichioides* strains to metconazole, prothioconazole, and tebuconazole were determined by a mycelial growth inhibition method, as described in previous studies [17,31]. All *Fusarium* isolates were derived from frozen (−80 °C) spore suspensions and grown on PDA medium for 2–3 days. Later, single cultures were transferred to new PDA plates. The 10 mm mycelial plug from the edge of seven-day-old *Fusarium* colonies was transferred face down to the centers of the prepared PDA plates containing different concentrations of fungicides. The treated plates were incubated at 25 ± 2 °C. The mycelial radial growth (mm) was measured by the crossing method [32] for 3 days until the fungus nearly covered the control plate. The inhibition caused by each fungicide concentration was expressed as a percentage value. We improved the formula [33] slightly by subtracting the diameter of the transferred mycelial plug for data normalization. The percent inhibition was calculated as follows (1):

Percent growth inhibition (%) = ((dc − dt)/(dc − 10)) × 100%,(1)

where dc—colony diameter of control plate (without fungicide), dt—colony diameter in fungicide treated plate, and 10—is the added 10 mm mycelial plug. 

The raw data of the experiment are reported in the Supplementary Materials (Table S1).

### 2.5. Determination of Fungicide Effective Concentration (EC50) and Statistical Analyses

**Figure 1:**
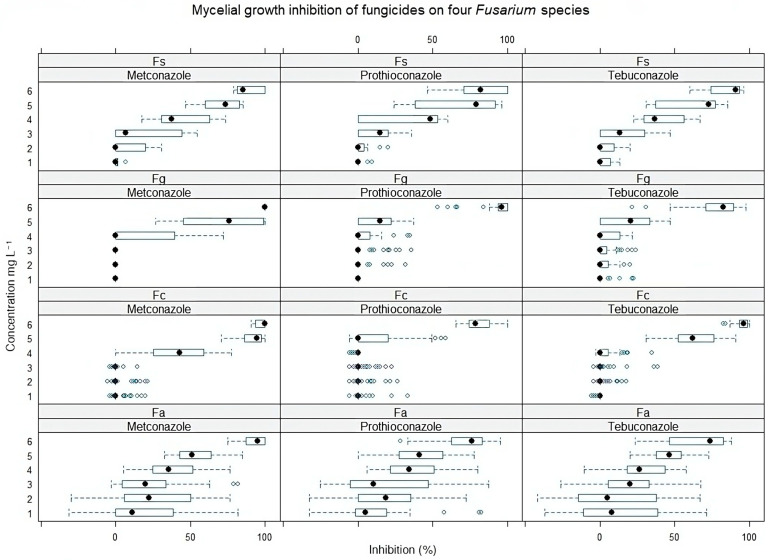
Mycelial growth inhibition (%) of four *Fusarium* species isolated from various plants at different concentrations of metconazole, prothioconazole, and tebuconazole. Fs—*F. sporotrichioides*, Fg—*F. graminearum*, Fc—*F. culmorum*, Fa—*F. avenaceum*. Concentrations: 1—0.00025, 2—0.0025, 3—0.025, 4—0.25, 5—2.5, 6—25 mg L^−1^. The black circle indicates the median inhibition values of all Fusarium isolates from the indicated host plant. The blue bar shows a range from the minimum to maximum values. Error bars indicate ± SE.

**Figure 2:**
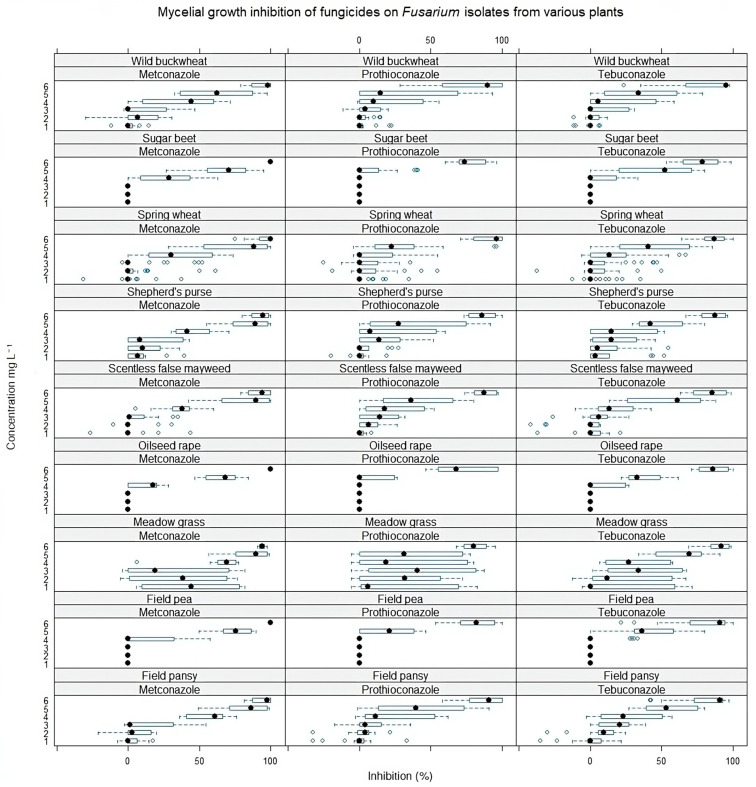
Mycelial growth inhibition (%) of all *Fusarium* isolates under the influence of different fungicides and their concentrations. The concentrations of fungicides indicated on the *Y*-axis are 1—0.00025, 2—0.0025, 3—0.025, 4—0.25, 5—2.5, 6—25 mg L^−1^. The black circle indicates the median inhibition values of all *Fusarium* isolates from the indicated host plant. The blue bar shows a range from the minimum to maximum values. Error bars indicate ±SE.

The effective metconazole, prothioconazole, and tebuconazole concentrations, which reduced mycelial growth by 50% (EC50 values), were determined for each *Fusarium* strain using the ‘drc’ (Dose–Response Curve) package [34] and nonlinear four-parameter log-logistic model (LL.4) [35] in R (version 4.2.3). One-way ANOVA was performed to determine the significance level of each fungicide’s EC50 to *Fusarium* isolates. The research data were processed by Tukey’s HSD (honestly significant difference) test (*p* = 0.05) to compare significant differences between isolates. Mean ± SEM (standard error of the mean) was used to describe the variability of measurements. Box-and-whisker plots were made using *geom_boxplot* function *ggplot2* library in R studio to visualize the data (Figure 1 and Figure 2).

## 3. Results

### 3.1. Mycelial Growth Inhibition Assay

**Figure 3:**
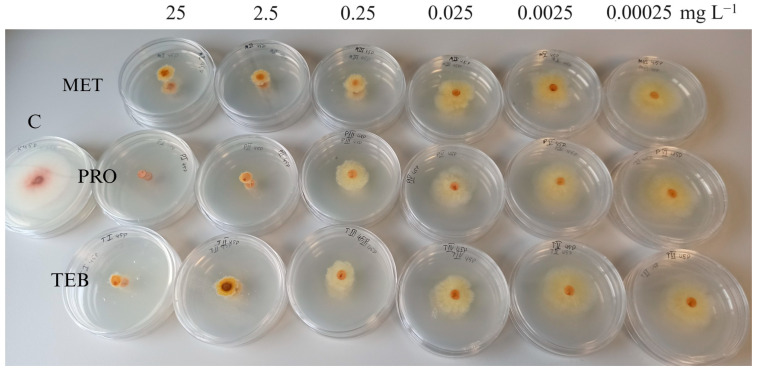
Mycelial growth inhibition of Fusarium sporotrichioides strain (45P) from wheat by different concentrations of metconazole (MET), prothioconazole (PRO), and tebuconazole (TEB). C—control plate without fungicides.

An in vitro sensitivity test of metconazole, prothioconazole, and tebuconazole for four different *Fusarium* species (*F. graminearum*, *F. avenaceum*, *F. sporotrichioides*, and *F. culmorum*) isolated from weeds, crops, and spring wheat showed different results. We noticed that *F. graminearum* and *F. culmorum* species, which usually cause FHB in Lithuania, were relatively less sensitive to triazoles than other species (Figure 1). Isolates of *F. avenaceum*, even at low doses, were effectively suppressed. However, negative inhibition values were also detected for this species, while the *F. sporotrichioides* isolates were shown to be the most sensitive to all triazoles (Figure 3).

Comparing the mycelial growth inhibition of four *Fusarium* species obtained from different plants, the least susceptible were the *Fusarium* isolates from sugar beet and oilseed rape plants (Figure 2). The *Fusarium* isolates from weed (shepherd’s purse, field pansy, and scentless false mayweed) differed in sensitivity and were least resistant to all fungicides. Also, we noticed that the susceptibility of isolates from meadow grass to fungicides did not vary gradually with concentration. The resistance of these weed isolates to fungicides ranged from weak to complete susceptibility, even at low doses.

### 3.2. Determination of EC50 Values for Fusarium Isolates

The estimation of the fungicide EC50 values for all *Fusarium* isolates revealed that metconazole was significantly more efficient in comparison to prothioconazole and tebuconazole. However, no differences were found in the EC50 values between prothioconazole and tebuconazole (Table 2).

Generally, the EC50 values of all isolates on metconazole were lower than 2.9 mg L^−1^, while prothioconazole EC50 ranged from 0.12 to 23.6 mg L^−1^ and tebuconazole ranged from 0.09 to 15.6 mg L^−1^ (Table 3, Table 4 and Table 5). The EC50 values for metconazole were 0.3–2.2 mg L^−1^ for *F. avenaceum*, 0.18–1.6 mg L^−1^ for *F. culmorum*, 0.18–2.9 mg L^−1^ for *F. graminearum*, and 0.05–1.9 mg L^−1^ for *F. sporotrichioides*. The EC50 values for prothioconazole were significantly higher: 0.12–16.8 mg L^−1^ for *F. avenaceum*, 2.4–21.4 mg L^−1^ for *F. culmorum*, 2.2–22.9 mg L^−1^ for *F. graminearum*, and 0.15–23.5 mg L^−1^ for *F. sporotrichioides*. The tebuconazole EC50 values ranged from 1.1 to 15.1 mg L^−1^ for *F. avenaceum*, 1.1–3.6 mg L^−1^ for *F. culmorum*, 2.6–25.6 mg L^−1^ for *F. graminearum*, and 0.09–5.4 mg L^−1^ for *F. sporotrichioides*.

Isolates 1P, 9P (*F. avenaceum*), and 33P (*F. graminearum*) obtained from weeds had a significantly higher mean of metconazole EC50 than the other isolates. The EC50 values recorded on prothioconazole were higher than on metconazole, except in the case of the 38P (*F. sporotrichioides*) and 7P (*F. avenaceum*) isolates. Prothioconazole showed the lowest growth inhibition of isolate 9P (*F. avenaceum*), 16P, 18P, 19P, and 21P (*F. culmorum*). However, the tebuconazole EC50 values did not show any significant difference between the isolates from weeds (*p* > 0.05). The isolates of *F. avenaceum* were less sensitive to metconazole than the isolates of *F. culmorum*, *F. graminearum*, and *F. sporotrichioides*. Meanwhile, four *F. culmorum* and one *F. avenaceum* isolate were the least susceptible to prothioconazole. In contrast, the *F. sporotrichioides* isolates were the most sensitive to all fungicides.

The average EC50 values of the *Fusarium* isolates obtained from crops were slightly higher than the values of those from weeds. In this case, the isolates of all *Fusarium* species were less sensitive to prothioconazole, and the EC50 varied from 2.9 to 23.6 mg L^−1^. The most effective fungicide was metconazole, with EC50 values ranging from 0.21 to 2.8 mg L^−1^. The *F. culmorum* isolates appeared to be the most sensitive to metconazole compared to the *Fusarium* species from crops. In contrast, the average prothioconazole EC50 values obtained from this species were the highest.

The *Fusarium* isolates from wheat were best inhibited by metconazole: the EC50 ranged from 0.05 to 2.9 mg L^−1^. Only 35P and 36P *F. graminearum* isolates on metconazole significantly differed from the others. The EC50 values of prothioconazole were the highest for the *F. culmorum* 23P isolate, while the most sensitive to this fungicide was *F. sporotrichioides* isolate 45P. The EC50 values for tebuconazole differed among the *Fusarium* species. The least sensitive were three out of five *F. graminearum* isolates, with EC50 values of 10.1, 11.4, and 16.2 mg L^−1^.

## 4. Discussion

We evaluated the effect of triazole fungicides on different *Fusarium* species isolated from weeds, crops, and spring wheat. Our study was based on the inhibition of *Fusarium* mycelial growth in vitro at six different concentrations of metconazole, prothioconazole, and tebuconazole. Metconazole was found to be the most efficient fungicide against *Fusarium*. This fungicide inhibited the mycelial growth of *F. graminearum* isolates from weeds and crops, namely, *F. culmorum* and *F. sporotrichioides* from crop plants, by 100% at a 25 mg L^−1^ concentration. However, metconazole at 25 mg L^−1^ showed a significantly weaker effect on *F. sporotrichioides*, *F. avenaceum*, and *F. culmorum* weed strains at 82%, 93%, and 94%, respectively. Similar to our findings, Ivic et al. [36] conducted a study that showed that metconazole at 10 mg L^−1^ inhibited *F. graminearum* growth by 100%, while tebuconazole at the same concentration exhibited an 87.8% inhibition. At low concentrations (0.00025–0.025 mg L^−1^), the *F. graminearum* species proved completely resistant to metconazole. These findings are consistent with several reports that have shown that the susceptibility of *Fusarium* to antifungal agents varies between species [37,38,39]. The mycelial growth of *F. graminearum* and *F. avenaceum* species was least inhibited by tebuconazole. These results corroborate the findings of Paul et al. [18] that demonstrated that metconazole and prothioconazole were significantly more effective for reducing FHB than tebuconazole. However, prothioconazole was less effective than tebuconazole for *F. sporotrichioides* strains from crops. Meanwhile, the growth of isolates of this species from weeds was most effectively inhibited by prothioconazole, followed by tebuconazole and metconazole. The growth of *F. culmorum* was most effectively inhibited by metconazole and tebuconazole. Additionally, it was observed that, in some cases, azoles more strongly inhibited the growth of *Fusarium* isolates from weeds than from crop plants. No data were found regarding the susceptibility of *Fusarium* strains isolated from alternative host plants (weeds) to fungicides. 

One interesting finding is that strains of *F. avenaceum* species were sensitive even to low metconazole concentrations. Some isolates were inhibited up to 82% at 0.00025 mg L^−1^, while others showed even better growth than the control. These findings demonstrate *F. avenaceum* species’ huge genetic variety with different levels of resistance to fungicides, and are in agreement with those of Falcão et al. [40] and Gaviria-Rivera et al. [41]. Surprisingly, negative inhibition was observed in the *F. avenaceum* isolate from spring wheat, whereas the lowest resistance (high growth inhibition) was found in *F. avenaceum* isolates from meadow grass. Recent studies [42] indicated that the most aggressive *F. avenaceum* isolate was the least fungicide-sensitive. In our case, the tests of *F. culmorum* and *F. avenaceum* from weeds’ sensitivity to fungicides showed that some isolates at 0.00025–0.25 mg L^−1^ had negative inhibition. This corroborates with other studies [43] suggesting that low concentrations of the active substance of fungicide complex (one of them being tebuconazole) stimulate the accumulation of *F. culmorum* fungal biomass compared to controls. Cendoya et al. [13] found that sub-lethal doses of fungicides can enhance not only *Fusarium* growth, but also the production of mycotoxins. It is believed that, under certain conditions, fungicides act as stress factors, inducing the production of mycotoxins until the growth of the fungus is inhibited [40,44].

The study of the effective concentration of the fungicide, which inhibits fungal mycelial growth by 50%, showed that the susceptibility varied among the isolates and species. *Fusarium* isolates were most sensitive to the fungicide metconazole: the EC50 values were lower than 2.9 mg L^−1^. The EC50 values for prothioconazole ranged from 0.12 to 23.6 mg L^−1^ overall, and for tebuconazole, from 0.09 to 15.6 mg L^−1^. In general, the fungicide acts very differently even in isolates of the same *Fusarium* species. For example, *F. graminearum* isolate 35P from spring wheat required a prothioconazole concentration of 14.7 mg L^−1^ to reduce its growth by 50%, whereas isolate 36P of the same species required only 3.4 mg L^−1^. In a US study, Anderson et al. [45] showed that 45 *F. graminearum* isolates collected between 2000 and 2014 had mean EC50 values of 0.0405 and 0.3311 mL L^−1^ for metconazole and tebuconazole, respectively. Our study obtained higher average values of these fungicides against *F. graminearum*: 1.478 mg L^−1^ for metconazole and 10.877 mg L^−1^ for tebuconazole. Similarly, Liuyuan et al. [46] showed that metconazole was the most effective among other seven fungicides (difenoconazole, epoxiconazole, carbendazim, phenamacril, pydiflumetofan, tebuconazole, and prothioconazole) in inhibiting the growth of *F. graminearum* with an average EC50 value of 0.032 mg L^−1^, whereas prothioconazole’s EC50 values were the highest (average 0.55 mL L^−1^). In our case, the EC50 values of prothioconazole for *F. graminearum* ranged from 2.2 to 22.9 mg L^−1^. This study showed that the *Fusarium* isolates from weeds (scentless false mayweed, field pansy, shepherd’s purse, meadow grass) were more susceptible to fungicides than the isolates from crops. The EC50 values of metconazole for *F. avenaceum* ranged from 0.3 to 2.203 mL L^−1^. Meanwhile, the EC50 values for *F. culmorum* (0.187–0.365 mL L^−1^), *F graminearum* (0.189–0.302 mL L^−1^) (except 33P), and *F. sporotrichioides* (0.057–0.455 mL L^−1^) were not statistically significantly different between weed species. Metconazole’s EC50 values obtained for isolates from crops (sugar beet, field pea, and oilseed rape) were higher: from 0.275 to 1.677 mL L^−1^ for *F. culmorum*, 1.9–2.812 mL L^−1^ for *F. graminearum*, and 0.852–1.9 mL L^−1^ for *F. sporotrichioides*. Generally, the *Fusarium* isolates from sugar beet and oilseed rape required higher concentrations of fungicides to inhibit all *Fusarium* species’ growth, whereas the lowest EC50 values were obtained with isolates from weeds. We speculate that *Fusarium* isolates residing in crops are less sensitive to fungicides due to the development of partial resistance due to their constant use on cultivated plants. According to de Chaves et al. [24], fungicide use on a broad scale has the potential to select resistant strains, which enhances the production of mycelium. Our hypothesis that isolates from spring wheat would be the least susceptible to fungicides due to their seasonal application on these plants was not confirmed. The EC50 values for the isolates from spring wheat were significantly lower than those from crop plants, but higher than or similar to those from weeds.

Our previous study [6] evaluated FHB disease severity, AUDPC, and 1000-grain weight in wheat infected with these *Fusarium* isolates, and results showed that *F. graminearum* isolate 5SP3p3-1 (30N) caused the highest FHB severity (70%). In our investigation, this most pathogenic isolate was the least sensitive to the fungicide tebuconazole (EC50 25.6 mg L^−1^) and had the second highest EC50 value for prothioconazole among all isolates. Meanwhile, the lowest severity of the disease was recorded in the *F. avenaceum* and *F. sporotrichioides* species, averaging 12.6% and 11.9%, respectively. According to our sensitivity test, we found that *F. sporotrichioides* and some isolates of *F. avenaceum* were the most susceptible to fungicides. From the aforementioned study, the 18P (CBP1401r) *F. culmorum* isolate from weed caused the highest FHB disease intensity and 1000-grain weight loss compared to the control. In our case, the 18P isolate had the highest EC50 values of all fungicides compared to the other *F. culmorum* isolates from weeds. Another highly pathogenic strain of *F. culmorum* (21P) required a high concentration of prothioconazole for growth inhibition with an EC50 value of 18.214 mg L^−1^. This suggests that highly aggressive strains require higher fungicide doses to reduce growth and are less sensitive to fungicides.

Our results indicate a partial development of resistance to triazole fungicides in *Fusarium* strains residing in cultivated plants compared to those found in weeds. Metconazole, prothioconazole, and tebuconazole are commonly used; however, we recommend avoiding the consecutive use of the same fungicides in the same field against these pathogens. Employing a strategy of mixing or alternating different fungicides can help mitigate the risk of developing resistant fungal strains [47]. We propose considering the use of metconazole alone or in combination with other fungicides to enhance the efficacy against Fusarium head blight (FHB) disease.

## 5. Conclusions

In conclusion, this study showed substantial differences in the sensitivity of *Fusarium* species isolated from different plants to metconazole, prothioconazole, and tebuconazole. We determined that *F. graminearum* and *F. culmorum* exhibit high resistance to triazole concentrations less than 2.5 mg L^−1^. Besides this, our data may be useful for understanding fungicides’ effect on the growth inhibition of different *Fusarium* species (isolated from weeds, crops, and spring wheat). However, this study was only conducted in vitro, so further field studies are needed to carry out susceptibility studies from the perspective of changing times and conditions.

## Figures and Tables

**Table 1:** Information on *Fusarium* isolates selected for sensitivity test to fungicides.

Isolate Number per Host Plant		Isolates per *Fusarium* Species
Weed		
Scentless mayweed	1 FG, 1 FC, 1 FA, 1 FS	*F. graminearum (FG)—13*
Shepherd‘s purse	1 FG, 1 FC, 1 FA, 1 FS	*F. culmorum (FC)—12*
Field pansy	1 FG, 1 FC, 1 FA, 1 FS	*F. avenaceum (FA)—6*
Wild buckwheat	1 FG, 1 FC, 1 FA, 1 FS	*F. sporotrichioides (FS)—9*
Meadow grass	1 FC and 1 FA	Total: 40 isolates
Crops		
Oilseed rape	1 FG, 1 FC, 1 FS	
Field pea	2 FG, 2 FC and 1 FS	
Sugar beet	2 FC, 1 FG, 1 FS	
Spring wheat	5 FG, 2 FC, 1 FA, 2 FS	

**Table 2:** Differences in effective fungicide concentrations that reduce mycelial growth by 50% (EC50) between *Fusarium* species, their source, and fungicides.

Source	Mean EC50 (mg L^−1^) ^1^	*Fusarium* Species	Mean EC50 (mg L^−1^) ^1^
Oilseed rape	8.851 a	*F. graminearum*	7.035 a
Sugar beet	8.075 a	*F. culmorum*	5.770 ab
Wild buckwheat	6.852 ab	*F. avenaceum*	4.194 bc
Field pea	6.762 ab	*F. sporotrichioides*	2.561 c
Spring wheat	4.205 bc	Fungicide	Mean EC50 (mg L^−1^) ^2^
Meadow grass	4.196 bc	Metconazole	0.952 b
Shepherd’s purse	3.291 c	Prothioconazole	9.315 a
Field pansy	3.229 c	Tebuconazole	8.315 a
Scentless false mayweed	3.078		

^1^ Means within column followed by different letters are significantly different according to Tukey’s HSD test at the *p* < 0.001 level. ^2^ Significance at *p* < 0.05 level.

**Table 3:** Effective concentration of metconazole, prothioconazole, and tebuconazole fungicides that reduces mycelial growth by 50% (EC50) for each *Fusarium* isolate obtained from weeds.

Isolate	Code	Source	*Fusarium* Species	EC50 (mg L^−1^) ^1^					
				Met	SEM	Pro	SEM	Teb	SEM
1P	TI1118c	*Tripleurospermum inodorum*	*F. avenaceum*	2.203 ab	1.936	7.672 b	16.309	4.886 a	5.549
4P	VA1110f	*Viola arvensis*		0.867 bc	0.559	4.918 bc	5.040	5.247 a	7.791
6P	CBP1149c	*Capsella bursa-pastoris*		0.307 c	0.205	1.834 bc	1.756	1.149 a	1.270
7P	PA1126s	*Poa annua*		0.949 bc	2.011	0.126 c	2.273	3.162 a	3.807
9P	FC1178fl	*Fallopia convolvulus*		1.925 ab	1.707	16.879 a	17.108	15.134 a	3.159
14P	TI1330r2	*Tripleurospermum inodorum*	*F. culmorum*	0.265 c	0.004	2.968 bc	0.092	1.197 a	0.034
16P	VA1164f	*Viola arvensis*		0.273 c	0.133	15.501 a	18.611	2.364 a	1.690
18P	CBP1401r	*Capsella bursa-pastoris*		0.365 c	0.053	18.868 a	33.419	3.691 a	0.154
19P	PA1129c	*Poa annua*		0.187 c	0.241	18.756 a	43.118	1.996 a	69.548
21P	FC1088r	*Fallopia convolvulus*		0.219 c	0.134	18.214 a	46.024	3.333 a	0.230
25P	TI1120c	*Tripleurospermum inodorum*	*F. graminearum*	0.302 c	0.009	5.808 bc	2.376	10.418 a	2.271
28P	VA541s	*Viola arvensis*		0.189 c	0.023	5.384 bc	64.189	3.636 a	2.224
29P	CBP1151f	*Capsella bursa-pastoris*		0.290 c	0.002	6.339 bc	0.166	6.194 a	1.490
33P	FC144r	Fallopia convolvulus		2.796 a	0.011	7.911 b	0.287	15.657 a	0.060
38P	TI1135l	*Tripleurospermum inodorum*	*F. sporotrichioides*	0.455 c	0.127	0.262 c	0.099	0.500 a	0.063
39P	VA1107f	*Viola arvensis*		0.057 c	0.026	0.154 c	0.030	0.164 a	0.026
42P	CBP1148f	*Capsella bursa-pastoris*		0.107 c	0.039	0.157 c	0.025	0.187 a	0.058
43P	FC1089c	*Fallopia convolvulus*		0.089 c	0.042	0.191 c	0.015	0.167 a	0.032

^1^ Means within the column followed by different letters differ significantly according to HSD test (*p* < 0.05). Met—metconazole, Pro—prothioconazole, Teb—tebuconazole, SEM—standard error of the mean. Letters in code means part of plant: roots (r), crowns (c), stems (s), leaves (l), florets (fl), and fruits (f).

**Table 4:** Effective concentration of metconazole, prothioconazole, and tebuconazole fungicides that reduces mycelial growth by 50% (EC50) for each *Fusarium* isolate obtained from crops.

Isolate	Code	Source	*Fusarium* Species	EC50 (mg L^−1^) ^1^					
				Met	SEM	Pro	SEM	Teb	SEM
14N	BN26r	*Brassica napus*	*F. culmorum*	0.871 ef	0.037	21.409 abc	0.091	2.399 a	0.075
16N	PS7r	*Pisum sativum*		1.677 cd	0.020	2.994 g	0.049	2.346 a	0.004
18N	PS23r	*Pisum sativum*		0.275 g	0.066	18.148 cde	0.187	1.740 a	4.958
20N	BV15.1l	*Beta vulgaris*		0.829 f	0.053	19.608 abcd	0.152	1.969 a	0.427
21N	BV18.1s	*Beta vulgaris*		0.212 g	0.024	18.988 bcd	0.165	1.412 a	0.323
27N	BN425l	*Brassica napus*	*F. graminearum*	2.140 b	0.010	15.889 de	0.006	5.945 a	0.173
28N	5PS3p3-2	*Pisum sativum*		1.912 bc	0.016	5.190 fg	0.045	4.259 a	0.137
30N	5PS3p3-1	*Pisum sativum*		2.144 b	0.043	22.909 ab	0.046	25.623 a	5.008
31N	BV7L6	*Beta vulgaris*		2.812 a	0.012	14.487 e	0.223	22.824 a	0.089
37N	BN9fl1	*Brassica napus*	*F. sporotrichioides*	1.938 bc	0.472	23.590 a	71.746	5.480 a	2.019
42N	PS37s	*Pisum sativum*		1.293 de	0.451	6.618 fg	1.206	4.304 a	1.848
43N	BV33.2s	*Beta vulgaris*		0.852 ef	0.230	8.708 f	2.172	4.199 a	1.745

^1^ Means within column followed by different letters differ significantly according to HSD test (*p* < 0.05). Met—metconazole, Pro—prothioconazole, Teb—tebuconazole, SEM—standard error of the mean. Letter in code means part of plant: roots (r), stems (s), leaves (l), florets (fl), pods (p).

**Table 5:** Effective concentration of metconazole, prothioconazole, and tebuconazole fungicides that reduces mycelial growth by 50% (EC50) for each *Fusarium* isolate obtained from main host-plant spring wheat.

Isolate	Code	Source	*Fusarium* Species	EC50 (mg L^−1^) ^1^					
				Met	SEM	Pro	SEM	Teb	SEM
23N	8SW5SP2	*Triticum aestivum*	*F. culmorum*	0.383 bc	0.005	2.406 def	0.003	1.190 cd	0.058
23P	8SWTG5SP4			0.217 bc	0.102	19.001 a	69.432	1.461 cd	0.576
34N	6SW4SP1		*F. graminearum*	0.325 bc	0.014	5.110 cde	1.092	11.402 ab	1.496
35N	6SW5SP1			0.312 bc	0.066	4.415 cde	8.570	10.155 ab	0.516
36N	6SW5SP19			0.224 bc	0.001	2.201 ef	2.677	2.674 cd	1.703
35P	6SW4SP1			2.796 a	0.011	14.718 b	0.169	6.383 bc	0.111
36P	6SW5SP1			2.967 a	0.017	3.396 cde	0.019	16.232a	0.213
45N	9SW5SP17-1		*F. sporotrichioides*	0.851 bc	0.124	5.281 cd	0.909	3.219 cd	1.281
45P	9SW5SP17-2			0.057 c	0.029	0.175 f	0.055	0.094 d	0.021
12P	8VK4V10		*F. avenaceum*	1.154 b	1.813	5.428 c	6.294	1.932 cd	14.249

^1^ Means within column followed by different letters differ significantly according to HSD test (*p* < 0.05). Met—metconazole, Pro—prothioconazole, Teb—tebuconazole, SEM—standard error of the mean.

## Data Availability

The data presented in this study are available on request from the corresponding author.

## Supplementary Materials

The following supporting information can be downloaded at: https://www.mdpi.com/article/10.3390/pathogens13020160/s1, Table S1: Mycelial radial growth and growth inhibition (%).
